# A Novel Approach for Optimizing Molecularly Imprinted Polymer Composition in Electrochemical Detection of Collagen Peptides

**DOI:** 10.3390/bioengineering12111272

**Published:** 2025-11-19

**Authors:** Naphatsawan Vongmanee, Jindapa Nampeng, Katesirin Rattanapithan, Phuritasinee Sriwichai, Chuchart Pintavirooj, Sarinporn Visitsattapongse

**Affiliations:** Department of Biomedical Engineering, School of Engineering, King Mongkut’s Institute of Technology Ladkrabang, Bangkok 10520, Thailand; naphatsawan.v@hotmail.com (N.V.); jindapa.na@kmitl.ac.th (J.N.); 65011329@kmitl.ac.th (K.R.); 65011464@kmitl.ac.th (P.S.); chuchart.pi@kmitl.ac.th (C.P.)

**Keywords:** collagen peptides, biosensors, biomolecules recognition, molecular imprinting technique, screen printed electrode, electrochemical measurement

## Abstract

Collagen peptides are key structural proteins that play an important role in maintaining the integrity and proper function of multiple tissues in the human body. Their breakdown is recognized as an important biomarker for various degenerative conditions, including the loss of muscle mass, joint and bone disorders, and compromised skin health. Current analytical approaches for collagen detection, such as ultraviolet spectrometry, enzyme-linked immunosorbent assay (ELISA), high-performance liquid chromatography (HPLC), and histochemical staining, are widely used but often expensive, time-consuming, and reliant on specific laboratory instrumentation, limiting their practicality for routine or rapid diagnostics. This study reports a novel biosensor for collagen peptide detection based on molecularly imprinted polymers (MIPs) integrated with screen-printed electrodes (SPEs). Electrochemical measurements revealed a clear correlation between collagen concentration and current response, confirming effective molecular binding within the imprinted matrix. The optimized MIP-modified electrode exhibited a detection range of 0.1–1000 µg/mL with a limit of detection (LOD) of 1.0106 µg/mL, limit of quantification (LOQ) of 4.46 µg/mL, sensitivity of 8.3816, and correlation coefficient (R^2^ = 0.9436). These results highlight strong selectivity and sensitivity toward collagen peptides. The proposed MIP-based biosensor provides a rapid, low-cost platform for detecting collagen degradation products and holds potential for early diagnosis and future clinical applications in degenerative disease monitoring.

## 1. Introduction

Collagen peptide complexes play a crucial role in maintaining cartilage structure and ensuring joint flexibility. The progressive degradation of these complexes is recognized as a key biomarker of several musculoskeletal disorders. In osteoarthritis (OA), cartilage breakdown leads to joint stiffness, discomfort, and restricted movement. Rheumatoid arthritis (RA), an autoimmune disease, is likewise associated with inflammation-driven cartilage loss. Furthermore, osteoporosis, a systemic skeletal disorder, involves deterioration of collagen integrity, contributing to weakened bone architecture [1,2,3]. With advancing age, collagen synthesis declines while its breakdown accelerates, resulting in a net loss of structural proteins within connective tissues [4]. This reduction in collagen levels contributes to several serious health issues in the elderly population, including autoimmune disorders that target collagen, such as rheumatoid arthritis, lupus, dermatomyositis, gout, and scleroderma [5,6,7,8]. Consequently, the ability to accurately monitor collagen peptide levels is essential for early disease detection and for guiding appropriate therapeutic strategies [9]. However, existing detection methods, including enzyme-linked immunosorbent assay (ELISA), high-performance liquid chromatography (HPLC), and mass spectrometry (MS) [10], suffer from significant limitations such as high cost, complex procedures, the need for trained personnel, and often inadequate sensitivity for reliable clinical application. These challenges hinder their integration into routine diagnostics and point-of-care systems.

To overcome these limitations, biosensors have emerged as a promising alternative owing to their high sensitivity, specificity, portability, and ease of operation [11,12,13,14]. Among them, electrochemical biosensors have gained considerable attention in biomedical research because of their rapid response, low production cost, and potential for miniaturization [15,16,17]. These devices have been widely utilized in diverse applications such as disease biomarker detection, tissue engineering, and drug discovery [18,19,20]. The analytical performance of electrochemical biosensors is largely determined by the surface modification of electrodes, which directly affects biomolecule immobilization efficiency and overall sensor functionality [21,22,23]. This study focuses on developing a novel biosensing platform based on molecularly imprinted polymers (MIPs) for the detection of collagen peptide complexes. MIPs serve as synthetic recognition elements designed to emulate natural antibody–antigen interactions, offering remarkable stability, selectivity, and cost-effectiveness. By harnessing this approach, the proposed biosensor aims to deliver high sensitivity and affordability, enabling timely detection and contributing to improved clinical outcomes through earlier intervention [9,24,25,26,27,28,29,30].

The methodology involves preparing a pre-polymer solution composed of monomers with amino functional groups, an optimized ratio of crosslinkers, glutaraldehyde as a bifunctional aldehyde-based crosslinker [31,32], an initiator, and a suitable solvent. Polymerization is conducted in the presence of a collagen peptide template, facilitating the formation of well-defined molecular cavities that are structurally complementary to the target peptides. These imprinted sites enable selective recognition and binding during the detection process. Optimization of polymerization conditions is essential, as excessive crosslinking or over-polymerization can distort the imprint structure and reduce binding efficiency [25,26,27,28,30]. Glutaraldehyde reacts with primary amine groups on the polymer surface to form imine linkages, which can further react with amine groups on the collagen peptide molecule. This two-step covalent bonding process facilitates stable immobilization of the protein, enhancing both sensitivity and long-term stability of the biosensor [33,34,35,36,37]. In addition, systematic investigations into template removal strategies were performed to enhance binding specificity and minimize non-specific interactions, which remain persistent challenges in MIP-based biosensors. Various conditions were optimized to maximize analytical performance across a collagen peptide concentration range of 0.1–1000 µg/mL. Careful optimization of these parameters ensures improved sensor performance in terms of selectivity, reproducibility, and operational stability. Collectively, this research presents an innovative MIP-based sensing approach designed to advance collagen peptide detection. The developed platform provides a practical and efficient analytical tool that may facilitate clinical diagnostics and enable long-term monitoring of degenerative diseases related to cartilage degradation and musculoskeletal health.

## 2. Materials and Methods

### 2.1. Sample Preparation of Collagen Peptides

A stock solution of collagen peptides was prepared by dissolving 1 mg of hydrolyzed collagen peptides (165044K, Sigma-Aldrich, St. Louis, MO, USA) in 1 mL of phosphate-buffered saline (PBS) pH 7.4 (524650, Sigma-Aldrich, St. Louis, MO, USA). Specifically, 1 mg of the collagen sample was accurately weighed using an analytical balance and transferred into a 1.5 mL Eppendorf tube. Subsequently, 1 mL of PBS was added, and the mixture was thoroughly homogenized using a vortex mixer. The solution was then gently warmed to 37 °C for 45 min to ensure complete dissolution. The resulting stock solution had a final concentration of 1 mg/mL (1000 µg/mL). This stock solution was further diluted with PBS to prepare working concentrations ranging from 0.1 to 1000 µg/mL, which were used in subsequent analyses throughout the study.

### 2.2. Polymers Synthesis for Collagen Peptides Detection

Polymers for collagen peptide detection were synthesized using four primary components to construct a selective polymer matrix. The first component was the monomer, consisting of hydroxyproline (H54409, Sigma-Aldrich, Darmstadt, Germany) combined with an amino acid standard (AAS18, Sigma-Aldrich, St. Louis, MO, USA), which is capable of selectively interacting with the target peptides. This monomer was designed to enhance the specific binding affinity toward the target protein. The second component was the initiator, azobisisobutyronitrile (AIBN) (441090, Sigma-Aldrich, Darmstadt, Germany), which was used to initiate the polymerization process. The reaction mixture was heated to 70 °C for 20 min to accelerate polymer formation. The third component was the cross-linker, N,N′-(1,2-dihydroxyethylene)bisacrylamide (DHEBA) (D5139, Sigma-Aldrich, Darmstadt, Germany), which was added to improve the mechanical rigidity and three-dimensional structure of the polymer. DHEBA also provides reversible cross-linking properties, allowing potential downstream recovery or modification of protein–polymer interactions. Additionally, glutaraldehyde (GA) (G6257, Sigma-Aldrich, Darmstadt, Germany) was introduced as an auxiliary cross-linking agent to further stabilize the polymer network and enhance peptide immobilization efficiency. GA reacts with amine-containing functional groups within the matrix, thereby improving the stability and durability of the imprinted sites. The fourth component was the solvent, dimethyl sulfoxide (DMSO) (D8418, Sigma-Aldrich, Darmstadt, Germany), a highly polar, aprotic solvent that is miscible with both water and a wide range of organic solvents. DMSO effectively dissolves the monomer, initiator, and cross-linker, ensuring homogeneous reaction conditions.

In this study, five polymer formulations were prepared by varying the ratio between the amino acid and glutaraldehyde, as shown in Table 1.

Next, AIBN was added as the initiator at a concentration of 1.5 mg (5 mg/mL) to each Eppendorf tube for all conditions, followed by the addition of 47 mg (156.7 mg/mL) of DHEBA as the cross-linker. GA was subsequently added at a fixed volume of 9.4 µL as an auxiliary cross-linking agent to further stabilize the polymer network and enhance peptide binding. DMSO was then added as the solvent at a volume of 300 µL to each tube. All components were thoroughly mixed using a magnetic stirrer, with the mixture heated on a hot plate at 80 °C for 30 min to initiate the polymerization process. After heating, the mixture was allowed to cool to room temperature, resulting in the formation of a complete pre-polymer gel solution.

### 2.3. Screen-Printed Electrode (SPE) Preparation

**Figure 1:**
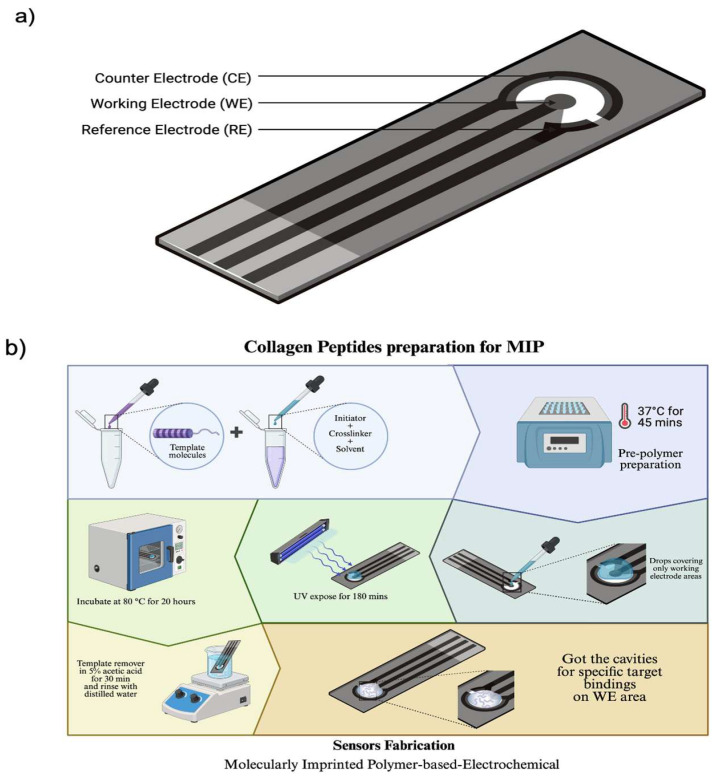
Diagram shows (**a**) 3 main types of Screen-Printed Electrodes, including Working, Reference and Counter Electrode. (**b**) The preparation of sensors.

A screen-printed electrode (SPE) (CI1703OR, Quasense, Bangkok, Thailand) used in this work, as shown in Figure 1, consists of three main components. First, the working electrode (WE) made of carbon serves as the sensing surface where oxidation and reduction reactions occur. Second, the reference electrode (RE), also made of carbon, provides a stable reference potential against which the potential of the WE is measured. Third, the counter electrode (CE), composed of Ag/AgCl, also known as the auxiliary electrode, allows current to flow and complete the electrochemical circuit.

The pre-polymer solution (described in Section 2.2) was carefully deposited onto the working electrode surface of each SPE, ensuring complete coverage. Five electrodes were prepared separately to represent each polymer formulation condition. The collagen peptide template of 1 mg/mL was then added onto the coated electrode. Subsequently, the electrodes were placed in a UVA chamber and exposed to UVA light at a wavelength of 365 nm for 3 h to initiate polymerization. The electrodes were then transferred to an oven and incubated at 80 °C for 20 h to complete the polymerization process. After polymerization, the collagen peptide template was removed by soaking the sensing surface of each electrode in 5% acetic acid for 30 min, followed by thorough rinsing with distilled water for another 30 min.

### 2.4. Cyclic Voltammogram Measuring

Cyclic voltammetry (CV) was performed to investigate the binding interactions between the molecularly imprinted polymers (MIPs) and the target analyte, collagen peptides. This technique involves applying cyclic potential to the screen-printed electrode and measuring the resulting changes in current response. Key aspects of the CV analysis include:

- Baseline Establishment: Initial CV scans were carried out on blank electrodes to determine background current levels.
- Binding Analysis: Upon introduction of collagen peptides, changes in the current response indicated successful molecular recognition by the MIPs.
- Calibration Curve Construction: The relationship between the percentage of relative current change and the logarithmic scale of collagen peptide concentration was plotted to evaluate sensor sensitivity and determine the detection limit.

The sample solutions were prepared by diluting the collagen peptides into five different concentrations, ranging from 0.1 µg/mL to 1000 µg/mL, using the following:

(1) $$C_{1}V_{1}=C_{2}V_{2}$$

where

$C_{1}$ = 1000 µg/mL (stock concentration);
$C_{2}$ = Desired concentration (µg/mL);
$V_{2}$ = 1000 µL (final volume);
$V_{1}$ = Volume of stock needed;
$V_{1}$ = $\frac{C_{2}\;\times \;V_{2}}{C_{1}}$.

In this work, a potentiostat (DRP-STAT400, Metrohm, Dropsens, Asturias, Spain) was connected to a computer and operated using the DropView 8400 software. The screen-printed electrodes prepared with different ratios of pre-polymer coating, as described in previous sections, were connected to an electrode analyzer for electrochemical measurements. Cyclic voltammetry (CV) was performed by scanning the potential from −0.4 V to +0.7 V at a scan rate of 50 mV/s. After baseline measurements were obtained, collagen peptide samples within the analytical concentration range were sequentially deposited onto the SPE surface, starting from the blank (without analyte) and increasing stepwise from 0.1 µg/mL to 1000 µg/mL. Each sample was prepared by diluting in the redox couple solution as a potassium ferri/ferrocyanide solution [Fe(CN)_6_]^3−^/[Fe(CN)_6_]^4−^. The resulting current responses were recorded, and the percentage of relative current change (%ΔI) was calculated. These values were then plotted against the logarithm of collagen peptide concentrations to construct a calibration curve.

### 2.5. Selectivity Test Preparation

To evaluate the selectivity of the sensor toward collagen peptides, gelatin was chosen as a structurally related interfering analyte. Gelatin (G9382, Sigma-Aldrich, St. Louis, MO, USA) was prepared at the same concentration levels used for collagen peptide measurements (0.1–1000 µg/mL) to ensure direct comparability. For each concentration, 100 µL of the gelatin solution was dropped onto the MIP-modified working screen-printed electrode. Cyclic voltammetry measurements were performed at room temperature using the polymer synthesized under the optimal conditions determined for collagen peptide detection.

## 3. Results

In this study, the current response corresponding to varying concentrations of collagen peptides was measured using a carbon screen-printed electrode (CI1703OR, Quasense, Bangkok, Thailand). Measurements were conducted under five different polymer synthesis conditions. The electrochemical responses obtained from each condition were compared to the assessment of the performance of the respective polymer compositions.

### 3.1. Cyclic Voltammogram of Carbon Electrode

**Figure 2:**
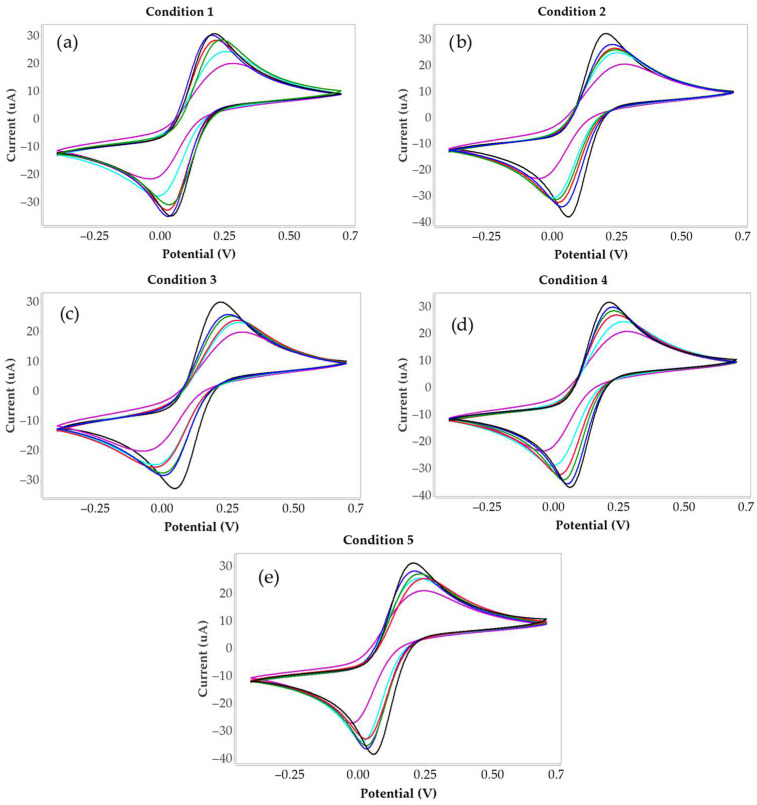
Cyclic voltammograms of collagen peptides at a scan rate of 50 mV/s within the potential window of –0.4 V to +0.7 V under different polymer synthesis conditions: (**a**) Condition 1; (**b**) Condition 2; (**c**) Condition 3; (**d**) Condition 4; and (**e**) Condition 5. The black line represents the blank electrode, while the blue, green, red, light blue, and purple lines correspond to collagen concentrations of 0.1 µg/mL, 1 µg/mL, 10 µg/mL, 100 µg/mL, and 1000 µg/mL, respectively.

**Figure 3:**
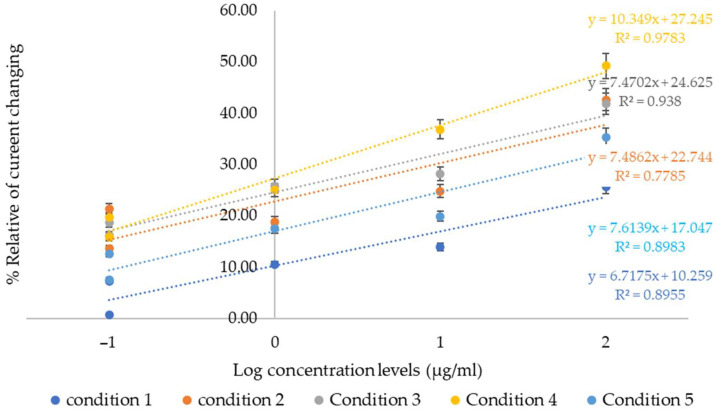
Comparison of the linearity range in five conditions of polymer synthesis to detect collagen peptides at any concentration levels on the carbon electrode. Data are presented as mean ± SD (repeated measurement *n* = 3).

The results present the current data obtained from the peak heights of the cyclic voltammograms, which were analyzed using the DropView 8400 software. The cyclic voltammetry results for all conditions are presented in Figure 2. The current of the blank sample was used as the baseline, and the current values of the collagen-containing samples were subtracted from the blank to calculate the current difference (ΔI). These ΔI values were subsequently used to determine the percentage relative current change (%ΔI), as summarized in Table 2. The calculated %ΔI values were then plotted to construct a calibration curve, as illustrated in Figure 3.

Cyclic voltammetry measurements of MIP-modified screen-printed electrodes under different polymer synthesis conditions are shown in Figure 2. In all cases, the redox peak currents decreased with increasing collagen peptide concentration, reflecting peptide binding to the polymer surface and partial blockage of electron transfer. Among the conditions, condition 4 exhibited the highest peak currents, the most distinct peak separation, and the clearest differentiation of CV signals across the collagen peptide concentration range (0.1–1000 µg/mL), suggesting optimal polymer morphology and highly accessible binding sites. The decrease in current arises from the formation of an insulating peptide layer on the polymer surface, which hinders electron transfer at the electrode interface. Therefore, the extent of CV current decrease directly reflects the amount of collagen peptide bound, providing a quantitative measure of molecular recognition at the polymer surface. The combined effects ratio of HYP and AA in the polymer network likely contributes to the superior analytical performance observed for condition 4.

In Figure 3, the linearity of the current responses obtained from carbon electrodes synthesized under five different polymer synthesis conditions is shown. These results, derived from cyclic voltammetry measurements (Figure 2a–e), are compared with the logarithmic concentration levels of collagen peptides. Each electrode was tested with a series of collagen peptide concentrations ranging from 0.1 µg/mL to 1000 µg/mL. The findings revealed variations in sensitivity and signal stability among the tested conditions.

Condition 4 exhibited the highest current response and demonstrated the most linear correlation between the logarithmic concentration of collagen peptides and the percentage of relative current change, indicating superior performance in terms of sensitivity and detection capability. The sensitivity, based on the slope of the calibration curve, was calculated as 8.3816 with a regression coefficient (R^2^) of 0.9436. The limit of detection (LOD) was calculated using the standard formula:

LOD=3σ/S

where σ is the standard deviation of blank measurement, and *S* is the slope of the calibration curve. The resulting LOD was 1.0106 µg/mL, indicating the lowest concentration at which the target analyte can be theoretically detected within the practical range. The limit of quantification (LOQ) was calculated using the following:

$$LOQ=\frac{10\sigma }{S}$$

The calculated result 4.46 μg/mL represents the lowest concentration at which the target analyte can be reliably quantified.

The practical detection range was observed from 0.1 µg/mL onward. This lower limit was selected because selectivity tests have not yet been performed, and measurements below 0.1 µg/mL may not reliably reflect target-specific signals. Selectivity tests are planned for future studies. In the present work, the focus was on identifying the polymer composition that provides the optimal response toward the target collagen peptide.

Conditions 2, 3, and 5 also produced moderate current increases with acceptable linearity, whereas Condition 1 yielded lower and less consistent responses. These differences may be attributed to variations in polymer composition and their interactions with the electrode surface and target analyte. Overall, Condition 4 was determined to be the most effective formulation for electrochemical sensing of collagen peptides using a carbon screen-printed electrode.

To evaluate the analytical performance of the developed sensor, the LOD and linear range were compared with those of previously reported collagen or peptide-based sensors. Table 3 summarizes the analytical parameters and key features of various reported methods alongside the present work. This comparison provides a broader context for assessing the sensitivity, detection range and practical advantages of the proposed MIP-based electrochemical sensor.

Compared with other simple collagen sensors reported in the literature [38,39,40,41], the present MIP-based electrochemical sensor offers a much broader linear range (0.1–1000 µg/mL) and excellent stability. Although the LOD is moderate (1.0106 µg/mL), the sensor is antibody-free, chemically stable, easily fabricated, and reusable, making it particularly suitable for samples with high collagen concentrations or rapid screening applications. In contrast, other simple sensors often suffer from narrow linear ranges, limited reproducibility, or complicated fabrication processes.

### 3.2. Selectivity Characterization

**Figure 4:**
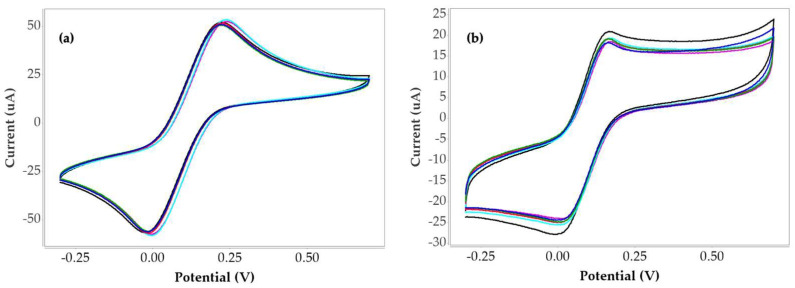
Cyclic voltammograms of collagen peptides and gelatine measured at a scan rate of 50 mV/s within the potential window of –0.4 V to +0.7 V under condition 4: (**a**) non-imprinted polymer and (**b**) gelatin measurement, The black line represents the blank electrode, while the blue, green, red, light blue, and purple lines correspond to collagen peptide and gelatin concentrations of 0.1 µg/mL, 1 µg/mL, 10 µg/mL, 100 µg/mL, and 1000 µg/mL, respectively.

To validate that the MIP sensor responds specifically to collagen peptides rather than to structurally related biomolecules, a selectivity assessment was carried out using gelatin as a potential interfering analyte. Gelatin was chosen because it originates from collagen and carries many of the same chemical features, including similar amino acid composition and functional groups that could, in principle, interact with the polymer network [42,43,44]. Although both materials share a comparable backbone, gelatin lacks the organized triple-helix structure of native collagen and displays a more disordered conformation with partially altered residue arrangements. These subtle but important structural differences make gelatin an appropriate challenge molecule for evaluating the sensor’s molecular recognition capability. To further demonstrate this selectivity, cyclic voltammograms of the non-imprinted polymer and gelatin were included for direct comparison, allowing clear visualization of the sensor’s baseline response and the minimal signal generated by the interfering analyte, as shown in Figure 4a and 4b, respectively.

**Figure 5:**
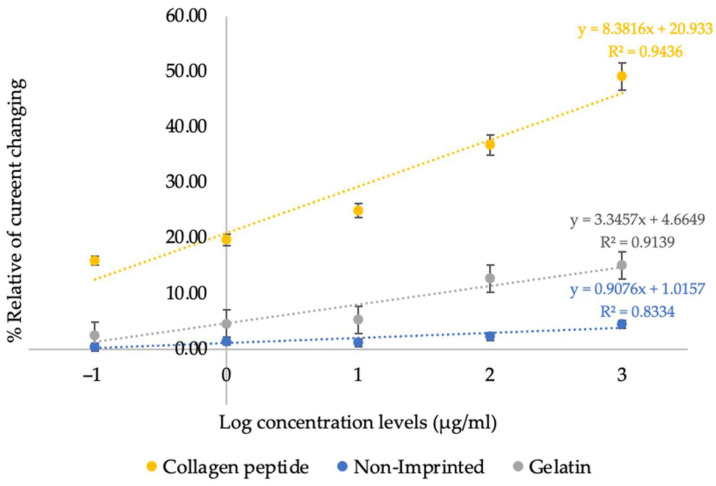
Comparison of the linearity range obtained from collagen peptide measurements, non-imprinted polymer, and gelatin detection. Data are presented as mean ± SD (*n* = 3).

In addition, a comparative calibration plot between collagen peptides, the non-imprinted polymer and gelatin measurement was constructed to highlight the markedly different signal intensities and linear responses among the three systems, as presented in Figure 5. The corresponding current values are summarized in Table 4.

The linear comparison shows that the sensitivities of collagen peptide, non-imprinted polymer, and gelatin measurements are 8.3816, 0.9076, and 3.3457, respectively. The corresponding coefficients of determination (R^2^) are 0.9436, 0.8334, and 0.9139. These results indicate that the polymer synthesized under condition 4 exhibits high specificity toward collagen peptides as reflected by its markedly greater sensitivity and strong linear correlation compared with the non-imprinted polymer and gelatin measurements.

In this study, the sensor’s response to gelatin was recorded under the same experimental conditions used for collagen peptides. The markedly lower signal obtained in the presence of gelatin, compared with the clear and concentration-dependent response observed for collagen peptides, confirms that the imprinting process successfully created selective binding sites. This outcome supports the sensor’s ability to distinguish the target peptides from closely related proteins and reinforces its suitability for practical applications.

## 4. Discussion

In this study, collagen peptide detection was performed using a functionalized carbon screen-printed electrode fabricated under different polymer synthesis conditions. The polymer coating was engineered by varying the ratio of HYP and AA to create an optimal surface for covalent immobilization and sensitive electrochemical detection. Among the tested conditions, the HYP to AA ratio of 2:3 (Condition 4) with GA as an auxiliary cross-linker demonstrated the best analytical performance, with the highest correlation coefficient (R^2^ = 0.9436) and the steepest calibration slope (8.3816), indicating excellent linearity and sensitivity across the analytical range. Cyclic voltammetry measurements under condition 4 exhibited distinct peak separation and well-defined current signals across the collagen peptide concentration range (0.1–1000 µg/mL). This superior performance can be attributed to the synergistic functions of each polymer component. HYP serves as the primary recognition unit, providing selective binding sites for collagen peptides through hydrogen bonding and specific interactions. AA acts as a co-monomer and structural modifier, influencing polymer porosity and flexibility. DHEBA functions as the main cross-linker, forming a stable polymer network, while GA serves as an auxiliary cross-linker that fine-tunes the spatial arrangement of binding sites and surface morphology. The optimized 2:3 HYP–AA ratio achieves a balance between abundant recognition sites and sufficient structural integrity, resulting in high surface accessibility and efficient electron transfer at the electrode interface, which enhances signal transduction and detection sensitivity. It should be noted that this work represents a preliminary investigation primarily focused on optimizing polymer surface modification parameters. Further studies are required to evaluate other critical biosensor performance metrics, including selectivity against other proteins and potential interfering biomolecules, to validate the sensor’s applicability in real biological samples. Overall, this study demonstrates a promising surface chemistry approach using a 2:3 ratio of HYP and AA polymer for developing a collagen peptide biosensor on carbon screen-printed electrodes. These findings provide a solid foundation for future developments, including sensor miniaturization, integration into point-of-care platforms, and clinical diagnostics applications.

## 5. Conclusions

This study aimed to develop a biosensor for collagen peptide detection using a surface immobilization strategy to enhance binding affinity and enable detection across a range of concentrations. Among the tested conditions, condition 4 on the carbon screen-printed electrode exhibited the best analytical performance, showing a high correlation coefficient (R^2^ = 0.9436), a steep calibration slope (8.3816), and a wide detection range (0.1–1000 µg/mL) with a limit of detection (LOD) of 1.0106 µg/mL by calculation and a limit of quantification (LOQ) of 3.36 µg/mL. These results highlight the sensor’s strong linearity and sensitivity for collagen peptide detection. However, further optimization and detailed characterization are necessary to improve the sensor’s selectivity, specifically toward collagen peptides. Future work will focus on identifying optimal monomers, cross-linkers, and polymerization conditions that could enhance both sensitivity and selectivity tests. Additionally, evaluating the biosensor over a wider range of collagen concentrations and in the presence of potential interfering substances will provide a more comprehensive understanding of its performance, robustness, and reliability in real-world applications.

## Figures and Tables

**Table 1:** Condition of polymer synthesis for collagen peptides detection.

Condition	Monomer Ratio (n:n)	HYP (mg)	AA (µL)	GA (µL)	DHEBA (mg)	AIBN (mg)	DMSO (µL)
1	1:1	13.1	8.2	9.4	47	1.5	300
2	1:4	13.1	32.8	9.4	47	1.5	300
3	3:1	39.3	8.2	9.4	47	1.5	300
4	2:3	26.3	24.6	9.4	47	1.5	300
5	2:5	26.2	41	9.4	47	1.5	300

**Table 2:** Summary of current results from carbon electrodes in all polymer synthesis conditions.

**Condition 1**	**Current (µA)**	**∆I (µA)**	**% Current changing**
Blank	32.74		
0.1 µg/mL	32.53	0.21	0.64
1 µg/mL	30.36	2.39	7.28
10 µg/mL	29.29	3.45	10.53
100 µg/mL	28.19	4.55	13.90
1000 µg/mL	24.34	8.40	25.66
**Condition 2**	**Current (µA)**	**∆I (µA)**	**% Current changing**
Blank	34.31		
0.1 µg/mL	29.66	4.66	13.57
1 µg/mL	26.99	7.32	21.34
10 µg/mL	27.83	6.48	18.88
100 µg/mL	25.80	8.51	24.79
1000 µg/mL	19.68	14.63	42.63
**Condition 3**	**Current (µA)**	**∆I (µA)**	**% Current changing**
Blank	31.46		
0.1 µg/mL	26.38	5.08	16.14
1 µg/mL	25.58	5.89	18.71
10 µg/mL	23.35	8.11	25.78
100 µg/mL	22.60	8.86	28.18
1000 µg/mL	18.31	13.15	41.80
**Condition 4**	**Current (µA)**	**∆I (µA)**	**% Current changing**
Blank	37.24		
0.1 µg/mL	31.33	5.91	15.88
1 µg/mL	29.91	7.33	19.68
10 µg/mL	27.93	9.31	24.99
100 µg/mL	23.53	13.71	36.81
1000 µg/mL	18.91	18.33	49.22
**Condition 5**	**Current (µA)**	**∆I (µA)**	**% Current changing**
Blank	32.86		
0.1 µg/mL	30.37	2.49	7.59
1 µg/mL	28.74	4.12	12.54
10 µg/mL	27.11	5.76	17.51
100 µg/mL	26.31	6.55	19.93
1000 µg/mL	21.27	11.59	35.27

**Table 3:** Comparison of the developed MIP-based electrochemical sensor with previously reported collagen peptide sensors.

Reference	Target	Sensor Type	LOD	Linear Range	SAMPLE MATRIX	Key Advantage/Remark
[38]	Collagen fragment	Colorimetric paper sensor	2 µg/mL	1–50 µg/mL	Buffer	Cheap, but very narrow linear range, not reusable
[39]	Collagen peptide	Simple electrochemical sensor	0.8 µg/mL	0.5–100 µg/mL	Buffer	Moderate LOD but linear range limited; electrode instability reported
[40]	Collagen type I	Optical sensor	5 µg/mL	2–80 µg/mL	Buffer	Easy readout, but fabrication complex and not reusable
[41]	Collagen I peptide	Paper-based electrochemical	3 µg/mL	1–100 µg/mL	Buffer	Portable, low cost, but low reproducibility and narrow working range
**This work**	Collagen peptide	MIP-based electrochemical	1.0106 µg/mL	0.1–1000 µg/mL	Buffer	Broad range, simple fabrication, antibody-free, stable, cost-effective

**Table 4:** Summary of current results from non-imprinted polymer and gelatin measurement.

**Non-Imprinted**	**Current (µA)**	**∆I (µA)**	**% Current changing**
Blank	52.38		
0.1 µg/mL	52.20	0.18	0.34
1 µg/mL	51.64	0.74	1.41
10 µg/mL	51.77	0.61	1.16
100 µg/mL	51.20	1.18	2.25
1000 µg/mL	50.05	2.34	4.46
**Gelatin**	**Current (µA)**	**∆I (µA)**	**% Current changing**
Blank	18.89		
0.1 µg/mL	18.44	0.45	2.40
1 µg/mL	18.02	0.87	4.58
10 µg/mL	17.89	1.00	5.29
100 µg/mL	16.49	2.40	12.71
1000 µg/mL	16.04	2.85	15.07

## Data Availability

The data presented in this study are available on request from the corresponding author. The data are not publicly available due to restrictions.
